# Introduction of an agent-based multi-scale modular architecture for dynamic knowledge representation of acute inflammation

**DOI:** 10.1186/1742-4682-5-11

**Published:** 2008-05-27

**Authors:** Gary An

**Affiliations:** 1 Department of Surgery, Northwestern University Feinberg School of Medicine, Chicago, IL, USA

## Abstract

**Background:**

One of the greatest challenges facing biomedical research is the integration and sharing of vast amounts of information, not only for individual researchers, but also for the community at large. Agent Based Modeling (ABM) can provide a means of addressing this challenge via a unifying translational architecture for dynamic knowledge representation. This paper presents a series of linked ABMs representing multiple levels of biological organization. They are intended to translate the knowledge derived from in vitro models of acute inflammation to clinically relevant phenomenon such as multiple organ failure.

**Results and Discussion:**

ABM development followed a sequence starting with relatively direct translation from in-vitro derived rules into a cell-as-agent level ABM, leading on to concatenated ABMs into multi-tissue models, eventually resulting in topologically linked aggregate multi-tissue ABMs modeling organ-organ crosstalk. As an underlying design principle organs were considered to be functionally composed of an epithelial surface, which determined organ integrity, and an endothelial/blood interface, representing the reaction surface for the initiation and propagation of inflammation. The development of the epithelial ABM derived from an in-vitro model of gut epithelial permeability is described. Next, the epithelial ABM was concatenated with the endothelial/inflammatory cell ABM to produce an organ model of the gut. This model was validated against in-vivo models of the inflammatory response of the gut to ischemia. Finally, the gut ABM was linked to a similarly constructed pulmonary ABM to simulate the gut-pulmonary axis in the pathogenesis of multiple organ failure. The behavior of this model was validated against in-vivo and clinical observations on the cross-talk between these two organ systems

**Conclusion:**

A series of ABMs are presented extending from the level of intracellular mechanism to clinically observed behavior in the intensive care setting. The ABMs all utilize cell-level agents that encapsulate specific mechanistic knowledge extracted from in vitro experiments. The execution of the ABMs results in a dynamic representation of the multi-scale conceptual models derived from those experiments. These models represent a qualitative means of integrating basic scientific information on acute inflammation in a multi-scale, modular architecture as a means of conceptual model verification that can potentially be used to concatenate, communicate and advance community-wide knowledge.

## Background

### The translational challenge arising from the multiple scales of biological organization

**Figure 1:**
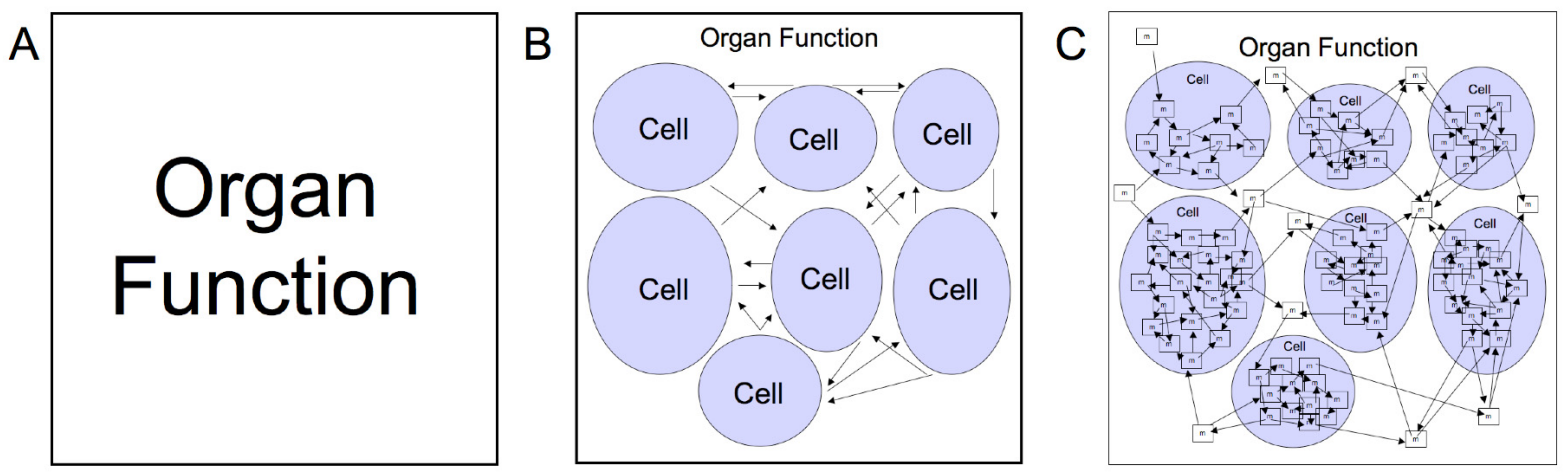
**Abstract demonstration of the expansion of information resulting from reductionist investigation of multi-scale biological systems**. Figure 1a shows the highest level of clinically observed phenomenon at the organ level. Figure 1b demonstrates graphically the mechanistic knowledge that organ function results from the interactions of multiple cells and types of cells. Figure 1c illustrates what a conceptual mechanistic model would look like when a further finer grained level of resolution is used. Figure 1c represents where the overwhelming bulk of biomedical research is currently being conducted, particularly with respect to the search for drug candidates and mechanisms of disease. Note that the "indistinctness" of Figure 1c is intentional: attempts to "zoom in" on the Figure may increase local clarity, but at the cost of being able to see the range of potential consequences to a particular manipulation.

The sheer volume of biomedical research threatens to overwhelm the capacity of individuals to process this information effectively, a situation recognized by the National Institutes of Health Roadmap in its "New Pathways" statement with its call for advancing integrative and multi-disciplinary research. Effective translational methodologies for knowledge representation need to move both "vertically" from the bench to the bedside, and be able to link "horizontally" across multiple researchers focused on different diseases. The hierarchical structure of biological systems is well recognized. Information is generated by research endeavors at multiple scales and hierarchies of organization: gene => protein/enzyme => cell => tissue => organ => organism. The existence of these hierarchies presents significant challenges for the translation of mechanistic research results from one organizational level to another (see Figures 1). The mirroring of these multiple levels in the organization of biomedical research has led to a disparate and compartmentalized community and resulting organization of data. The consequences of this are seen primarily in attempts to develop effective therapies for diseases resulting from disorders of internal regulatory processes. Examples of such diseases are cancer, autoimmune disorders and sepsis, all of which demonstrate complex, non-linear behavior. In particular, there has been growing interest in the study of inflammation as a common underlying mechanism in disease processes ranging from sepsis to atherosclerosis (as noted by the recent addition of inflammation as an Emphasis Area to the NIH Roadmap for Medical Research). The investigation of such a ubiquitous process presents significant challenges in the integration and concatenation of research efforts in both the "vertical" and "horizontal" directions.

### A possible solution: dynamic knowledge representation via agent-based modeling

Mathematical modeling and computer simulation offer a translational method for achieving this goal. More specifically, computer modeling can be seen as a means of dynamic knowledge representation that can form a basis for formal means of testing, evaluating and comparing what is currently known within the research community. In this context, the use of computational models is considered a means of "conceptual model verification," in which mental or conceptual models generated by researchers from their understanding of the literature, and used to guide their research, are "brought to life" such that their behavioral consequences can be evaluated. I propose that this use for computational models can be accomplished with relatively coarse-grained qualitative models. The justification for this belief is the fact that biological systems are generally robust. They function within a wide range of conditions, yet retain, for the most part, a great degree of stability with respect to form and function. A great reliance on minute specific parameters, particularly given the limitations of the capability for measurement, would connote a degree of "brittle-ness" in biological systems that is not substantiated by general observation. Furthermore, there are perpetual and unavoidable limitations with respect to the comprehensiveness with which a system can be quantitatively described; there will always be a degree of "incompleteness" in the knowledge of a biological system. Therefore, conceptual models will always be, to some degree, qualitative, and this fact should not preclude the use of computational methods to improve upon the current methods of representing (via graphs, diagrams and flow charts) and testing of these models.

**Figure 2:**
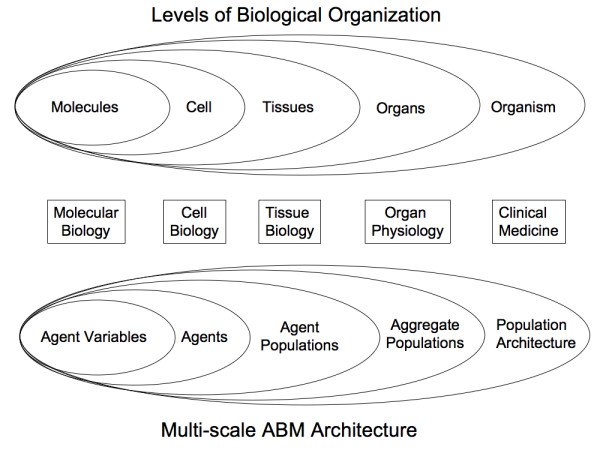
**Multiple scales of Biological Organization, Biomedical Research and Multi-scale ABM Architecture**. Representation of the multiple scales of biological organization and the ABM architecture in a nested fashion, to reflect the reliance of the higher scale behavior on the mechanisms operating at the lower levels. Of note, the biomedical research community structure in the middle is not so represented, to reflect the relative compartmentalization of the community with respect to the operational aspect of research, though obviously lower scale knowledge and information does influence the hypotheses generated and being tested at the higher scale.

Agent Based Modeling (ABM) is a computational modeling technique that is object-oriented, rule-based, discrete-event and discrete-time. ABM has characteristics that make it well suited for the goal of dynamic knowledge representation and conceptual model verification. The structure of ABM facilitates the development of aggregated modular multi-scale models [1,2]. ABM are based on the rules and interactions between the components of a system, simulating them in a "virtual world" to create an in-silico experimental model [3-7]. ABMs have been used to study biomedical processes such as sepsis [5,6], cancer [2,8], inflammatory cell trafficking [9] and wound healing [10]. They have an intrinsically modular structure via the grouping of components ("agents") into classes based on similar rules. ABM rules are often expressed as conditional statements ("if-then" statements), making ABM suited to expressing the hypotheses that are generated from basic scientific research. Individual agents "encapsulate" mechanistic knowledge in the form of a set of rules concerning a particular component. The importance of this "encapsulation" in ABM (as opposed to the "compressed" representation of knowledge with a mathematical formula, such as a biochemical rate law) is the placement of the mechanistic knowledge within a compartmentalized object. Furthermore, ABM goes beyond the mere instantiation of this knowledge as a single case by concurrently generating multiple instances of a particular "encapsulation/object." Because of this property, ABM is an expansion of mere rule-based and object-oriented methods. Multiple individual instances have differing initial conditions by virtue of existing in a heterogeneous environment. Because stochastic components are embedded in their rule systems (a well recognized property of biological objects [11-13]), individual agents have differing behavioral trajectories as the ABM is executed. This results in population-level dynamics derived from the generation of these multiple trajectories, population dynamics that, when viewed in aggregate, form the nested, multi-scalar/hierarchical organization of biological systems. In this fashion, ABM performs the trans-hierarchical function desired in an integrative modeling framework (Figure 2).

ABM, however, is not without its limitations. Specifically, two major limitations affect its use as a multi-scale modeling platform. The first has to do with the "black box" quality of ABM. Since the models rely on an ill-defined principle of "emergence" in order to transcend the epistemological boundaries represented by the multiple hierarchies of system organization, their behavior is difficult to characterize analytically. Therefore, ABMs are not "mathematical models" per se, being able to be subjected to formal analysis and "solved." Rather, the use of ABM falls into the category of "simulation science," in which computational analogs of real world systems are produced and used in a fashion similar to traditional experimental preparations. As such, the sizes of the models, in terms of numbers of components and scope of their environment, must have the extensibility at least to approach the dimensions of their real-world reference systems, particularly when multi-scale phenomena are the goal. Analytical tasks such as parameter sensitivity analysis and behavior-space determination rely upon brute force computation to generate data sets dense enough for appropriately grained statistical analysis. This requirement leads to the second hurdle in the use of ABM in a multi-scale context: their relatively high computational requirements as compared to equation based models. Currently, in general, most ABM platforms run as emulated parallel processing systems based on a single threaded central processing unit. The execution of an ABM requires multiple iterated computations as each discrete event is carried out, many more than for equation-based simulations, resulting in significantly greater computational demands. Despite ongoing work on hardware and software configurations to increase the computational efficiency of running ABMs, currently computational costs constrain the size of feasible ABM implementation. There is ongoing work in the development of "hybrid" model systems intending to use equations to model those aspects of a system in which mean-field approximations are valid, and link these components to ABMs where spatial heterogeneity and it effects are significant [14,15]. Additionally, methods are being developed to algorithmically increase the efficiency of the evaluation and analysis of complex multi-scale models [15]. This topic will be explored further in the Discussion.

These challenges notwithstanding, a modular multi-scale architecture using the agent-based paradigm is proposed in this paper. I believe the benefits of an agent-based architecture in terms of modularity, translational efficacy and structural/organization mapping to biological systems outweigh the current limitations of this technique. Furthermore, the case will be made that, in terms of effective knowledge representation, a qualitative approach may often suffice for the goal of conceptual model verification. Acute inflammation, as a ubiquitous multi-factorial example of biocomplexity, is used as the demonstration platform for a series of ABMs developed at multiple levels of resolution, extending from intracellular signaling leading up to simulated organ function and organ-organ interactions. Specifically, the model reference system is the clinical manifestation of multi-scale disordered acute inflammation, termed systemic inflammatory response syndrome (SIRS), multiple organ failure (MOF) and/or sepsis. These clinical entities form a continuum of disseminated disordered inflammation in response to severe levels of injury and/or infection, and represent one of the greatest clinical challenges in the current health care environment. The core of agent-based architecture is a "middle-out" approach that focuses on representing and modeling cellular behavior as the agent level. Cells form a natural choice for the agent level in an ABM architecture. Cells are categorized by type, based on discovered and hypothesized rules of behavior, and can, to a great degree, be treated as "input-output" devices acting within a local environment. Cells are structurally and functionally aggregated into tissues and organs, the overall behaviors of which are determined by the actions and interactions of their constituent cells. Furthermore, the bulk of ongoing biomedical research is aimed at affecting the behavior of specific cellular types by the manipulation of their internal rules, and it is exactly the translation of this type of information/knowledge beyond the realm of solitary cells that underlies the core need for a multi-scale modeling platform.

Therefore, the initial design aspects of a multi-scale architecture for modeling acute inflammation hinge upon identifying the key actors involved, and determining existing hypotheses aimed at unifying the problem of disseminated disordered inflammation. Two such unifying hypotheses involve viewing disordered systemic inflammation as either a disease of the endothelium [16-18] or a disease of epithelial barrier function [19]. The former paradigm points to the endothelial surface as the primary communication and interaction surface between the body's tissues and the blood, which carries inflammatory cells and mediators. Factors supporting this view are the fact that endothelial activation is a necessary aspect of the initiation and propagation of inflammation, particularly in the expansion of local inflammation to systemic inflammation, and that the histological and functional consequences of inflammation are extremely pronounced at the endothelial surface [17]. On the other hand, there is also compelling evidence that organ dysfunction related to inflammation is primarily manifest in a failure of epithelial barrier function. Pulmonary, enteric, hepatic and renal organ systems all display epithelial barrier dysfunction that has consequences at the macro-organ level (impaired gas exchange in the lung, loss of immunological competence in the gut, decreased synthetic function in the liver and impaired clearance and resorptive capacity in the kidney) [19]. The multi-scale architecture presented herein attempts to reconcile these two hypotheses by concatenating their effects within the design of the architecture: there is an epithelial barrier component that is used to represent the consequence of individual organ failure, and an endothelial/inflammatory cell component that provides the "binding" interaction space that generates, communicates and propagates the inflammatory response. The primary cell classes in this architecture are endothelial cells, blood borne inflammatory cells (with their attendant sub-types) and epithelial cells. The development outlined herein will progress start from ABMs representing the basic cell systems with essentially linear knowledge translation from basic science experimental data. The next step proceeds in a more abstract and qualitative fashion, extending to tissue/organ level ABMs that combine the constituent cell system models. It is at this step that the tissue/organ ABM becomes a dynamic instantiation of the epithelial-endothelial hypothesis mentioned above. The abstraction of the model centers on representing the "active" components involved in that hypothesis. The model will be validated by comparing its behavior to that of in-vivo organ-directed experiments using the established pattern oriented method described by Grimm et al. [20]. This method centers on the comparison, at multiple levels ranging from constituent rules to various observed phenomenological behaviors, between the model and the real-world reference system. Finally, the next level of biological organization will be represented by a multi-organ ABM that simulates the organ-level crosstalk seen in clinical situations. This model will be an abstract instantiation of the hypothesis linking the gut to the lung in the pathophysiology of MOF [21-23]. The qualitative nature of the latter two model levels is acknowledged. However, I wish to note that these models are presented as the initial manifestations of an evolvable multi-scale modeling architecture, a "blueprint" of a modeling framework that will be built upon in the future. Furthermore, despite the qualitative nature of the "scale-up" translation in these models, they do capture and instantiate the "essence" of specific pathophysiological hypotheses. The test of plausibility of these hypotheses (and note, the focus is on plausibility, not proof) can be examined through the behavior of these models and matching them to observations of equivalent scale experimental/clinical phenomena.

## Methods

### Development of the basic cell ABMs

The base endothelial/inflammatory cell ABM has been previously developed and described [5,6]. The following section will describe the development of the epithelial barrier model (epithelial barrier agent based model = EBABM). This development focuses on translating particular molecular pathways in a particular cell type: tight junction protein metabolism and pro-inflammatory signaling as pertaining to gut epithelial barrier function seen in the enterocyte component of the gut. Calibration and validation follow the established pattern oriented method well described for ABM [5,6,20] and consist of comparing the behavior of the model with in vitro reference model data.

### Reference model for the EBABM and validation experiments

**Figure 3:**
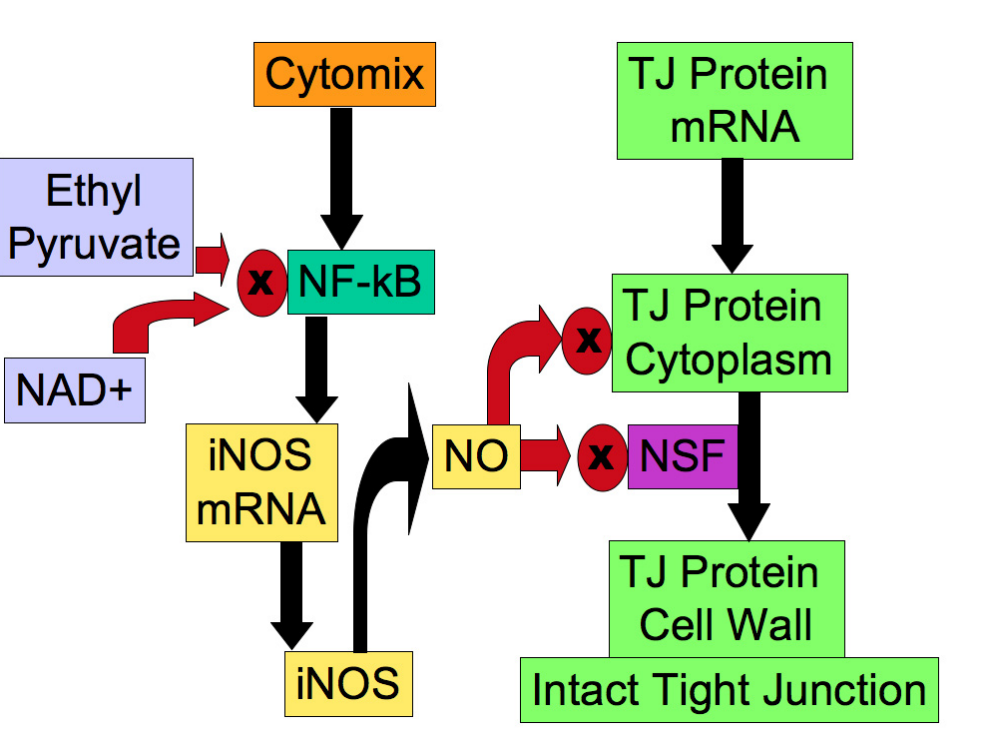
**Graphical Representation of the control logic extracted from the basic science references [24, 26, 27]on Gut Epithelial Barrier Function**. General flowchart of the components and mechanisms of TJ protein synthesis and localization, the effects of pro-inflammatory stimulation, and the effects of interventions with ethyl pyruvate and NAD^+^. All labeled boxes correspond to agent or environment state variables within the EBABM. In the actual code of the EBABM there are distinct pathways for the different TJ proteins (not shown here for clarity purposes).

The reference model for the EBABM is a well-described human cultured enterocyte model (Caco-2) and its responses to inflammatory mediators including nitric oxide (NO) and a pro-inflammatory cytokine mix ("cytomix") that includes tumor necrosis factor (TNF), interleukin-1 (IL-1) and interferon-gamma (IFN-gamma) [24-26]. These papers suggest that enterocyte tight junction (TJ) proteins are involved in the integrity of gut epithelial barrier function, and that the production and localization of TJ proteins are impaired in a pro-inflammatory cytokine milieu. The TJ proteins that seem to be most affected in this situation are occludin, claudin-1, ZO-1 and ZO-3. The primary mechanism proposed is the activation of nuclear factor kappa-B (NF-kappa-B) by pro-inflammatory cytokines leading to subsequent activation and production of inducible nitric oxide synthetase (iNOS). The nitric oxide (NO) produced inhibits synthesis of occludin, ZO-1 and ZO-3 while increasing production of claudin-1. Furthermore, the NO impairs localization of synthesized occludin, claudin-1 and ZO-1 to the cell surface. This effect appears to be due to the interference of NO with N-ethylmaleimide-sensitive factor (NSF), a molecule needed for localization of TJ proteins to the cell membrane [27]. These effects are seen both with administration of exogenous NO, and through intrinsic production via the cytomix-NF-kappa-B-iNOS pathway. These papers go on to investigate the effects of certain blocking agents. Addition of a NO scavenger [26] eliminates the effects of exogenous NO and cytomix. Administration of ethyl pyruvate [24] and nicotinamide adenine dinucleotide (NAD^+^) [25] both thought to inhibit NF-kappa-B, also both attenuate the effects of cytomix. Data points for levels of NO, TJ protein expression and permeability were at 12, 24 and 48 hours in all the experiments. Figure 3 is a graphical representation of the general control logic underlying the agent rule systems based on the knowledge extracted from [24-27].

### EBABM: construction and calibration

The EBABM was constructed using the freeware software toolkit Netlogo [28]. The architecture and rule systems for the ABM were constructed using the information gleaned from the papers listed above. The procedure for developing ABMs in the context of medical research has been extensively described [5,6] and critical points of development and structure will be summarized here.

**Figure 4:**
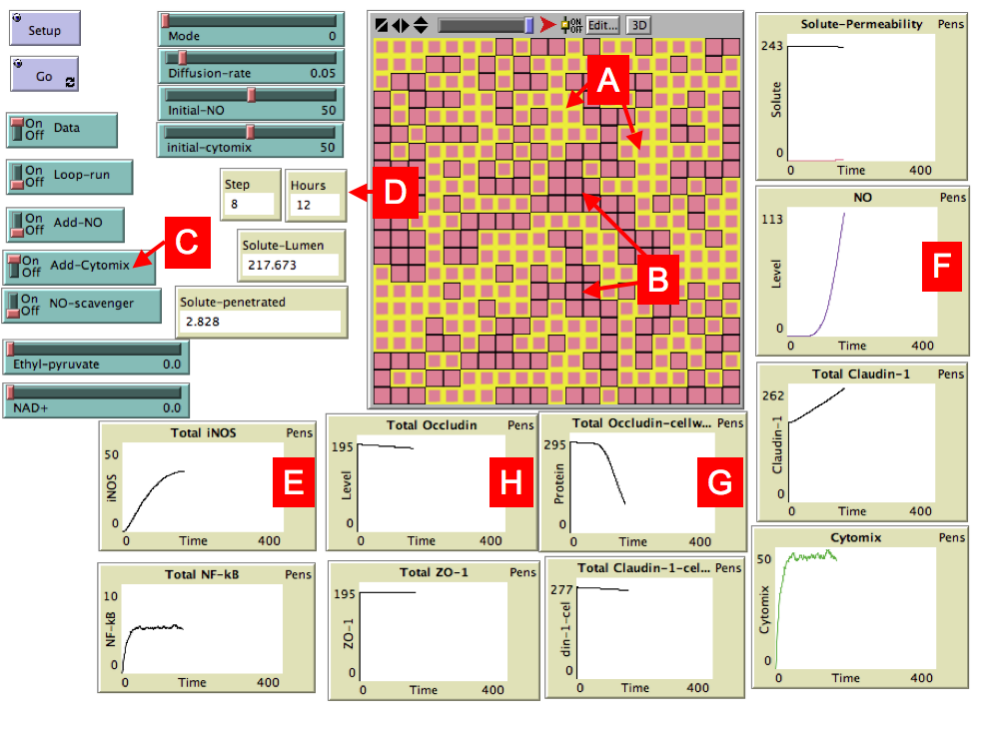
**Screen shot of the Graphical User Interface of the EBABM**. Control buttons are on the Left; Graphical Output of the simulation is in the center. Graphs of variables corresponding to levels of mediators and tight junction proteins are at the bottom and right. In the Graphical Output Caco-2 agents are seen as pink squares, those with intact Tight Junctions bordered in yellow (Letter A), those with failed Tight Junctions bordered in black (Letter B). This particular run is with the addition of cytomix (Letter C), seen after 12 hours of incubation (Letter D). The heterogeneous pattern of tight junction failure can be seen in the Graphical Output. Levels of Caco-2 iNOS activation can be seen in Graph Letter E, and produced Nitric Oxide (NO) can be seen in Graph Letter F. Of note, the total amount of tight junction protein occludin does decrease slightly (Graph Letter G), but the amount of occludin localized in the cell membrane drops much more rapidly (Graph Letter H), reflecting the impairment of occludin transport due to NO interference with NSF and subsequent loss of tight junction integrity.

The topology of the EBABM is a 2-dimensional square grid. The grid has 21 × 21 cells, in each of which there is an epithelial cell agent ("epi-cell'). The size of this grid was chosen as a representative portion of a total cell culture surface for reasons of computational efficiency; the processes being modeled by the EBABM are proportional to the cell surface area and the model could be, if desired, scaled up to any size. There are also two additional simulation "spaces," one layer representing the apical extracellular space (from which the diffusate originates) and another layer representing the basal extracellular space (into which the diffusate flows if there is permeability failure). A screenshot of the EBABM during an experimental run can be seen in Figure 4. Each epi-cell has 8 immediate neighbors, and at each contact point there is a simulated tight junction (TJ). The integrity of the TJ requires both apposed epi-cells to have adequate production and localization of TJ proteins. The epi-cell agent class contains variables that represent the precursors, cytoplasmic levels and cell membrane levels of the TJ proteins, as well as intracellular levels of activated NF-kappa-B and iNOS mRNA. Furthermore, there are "milieu" variables that represent NO, cytomix and the diffusate. Algorithmic commands were written for the synthesis of TJ proteins as well as the pathway for NO induction. Since ABM is a discrete event computational method, the updating of variables occurs via multiple iterations as the model is executed. Therefore there are no kinetic equations per se for the metabolic pathways modeled by the agent rules. Rather, the metabolic rules consist of a simple arithmetical relationship based on the prior state (value) of a particular variable used to calculate the current value. The specifics of the algebraic relationship (such as constant values) are tuned during the calibration process by comparing the values over time of the simulation variables against the reference data sets. While this method lacks the "precision" of formally measured and characterized kinetic rate equations, several factors support its use in this context. First are the purely pragmatic reasons; detailed metabolic kinetic data are difficult to obtain, do not exist for vast majority of metabolic processes (such as TJ protein metabolism), and even if obtained using ex vitro methods, may not reflect the kinetics present in an intracellular environment [29]. Additionally, we return to the concept of cells as robust dynamic objects, in which qualitative scaling of intracellular processes may actually be more than sufficient given the stochasticity observed in their dynamics [30]

Calibration of the model was done using three behavior patterns of the EBABM compared to observed phenomena in the reference experimental systems. The first calibration was for the basal diffusion rate. The diffusion coefficient in the unperturbed system was adjusted to match the rate of diffusion in the reference data set at times 12, 24 and 48 hours. This established the baseline control permeability. The second calibration was done to reproduce the levels of administered cytomix and NO. The reference data sets were the levels of measured NO in both the exogenous NO donor arm and the cytomix administration arm (as seen in Figure 1 from Ref [26]). Calibration occurred by modifying the coefficients of the NO induction pathway algorithm. The third calibration was done with respect to the TJ protein synthesis/breakdown algorithms. Steady state TJ protein levels were established using the inhibition data extrapolated from the Western Blot results from Ref [26]. For the purposes of this model, at this point in development of methods for model construction, calibrations in this section were done by hand, using trial and error. It is expected that in the future automated calibration algorithms would need to be developed in order to scale up this methodology to more extensive and detailed models.

Following these three levels of calibration the baseline EBABM was established. Note that this includes the EBABM perturbed with both NO and cytomix. No further modifications were done to the internal metabolism algorithms of the epi-cell class; the only additions were the presumptive metabolic effects of ethyl pyruvate and NAD^+ ^in the simulated experiments from Figure 6 from Ref [24] and Figure 2 from Ref [25], respectively.

**Figure 6:**
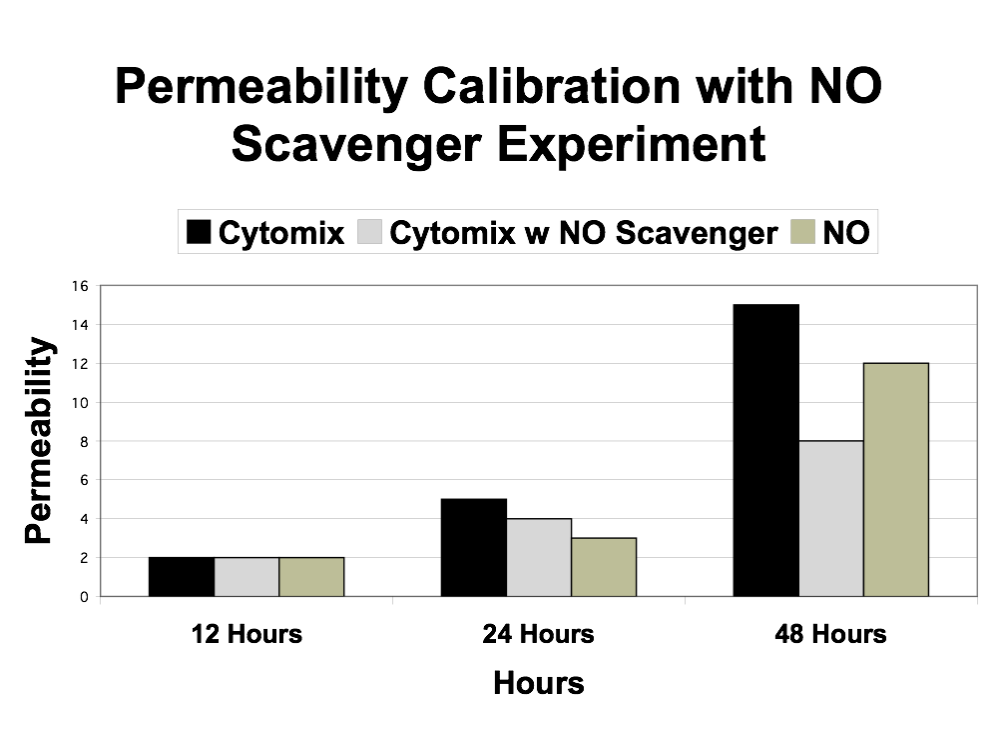
**Simulated Permeability to NO, Cytomix and Cytomix + NO scavenger**. Graph of calibration data of the permeability effects of NO and Cytomix, representing the diffusion rate through a failed epithelial barrier and the effect of NO on the algorithms for epi-cell TJ protein synthesis/localization. As with Figure 4, the black bars (= Cytomix) and beige bars (= Exogenous NO) are the calibration arms. This graph can be compared with the lower panel of Figure 1 in Ref [26].

### EBABM: simulations and results

There were three simulated interventions to the baseline EBABM: 1) addition of a NO scavenger [26], 2) addition of ethyl pyruvate [24], and 3) addition of NAD^+ ^[25]. The NO scavenger was simply modeled by reducing the level of the NO milieu variable after production. Both NAD^+ ^and ethyl pyruvate were modeled using their presumptive mechanisms of NF-kappa-B inhibition [25,31] by their insertion as negative influences in the NO induction pathway algorithm. In-silico experiments were run using these interventions with data points at 12, 24 and 48 hours as per the reference papers. Data collection looked at permeability reflecting TJ integrity, levels of TJ proteins and localization of TJ proteins.

**Figure 5:**
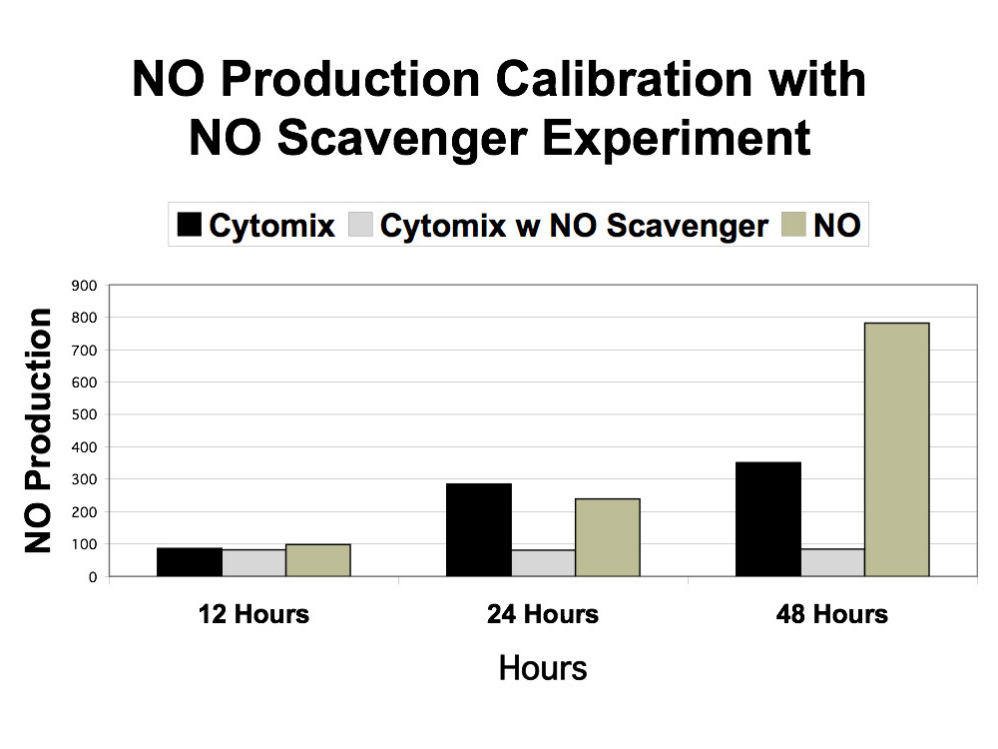
**Simulated Nitrogen Oxide (NO) Production and response to NO Scavenger**. Calibration data is seen in the black bars (= Cytomix) and the beige bars (= NO) with respect to simulation rules for NO production. The NO data match the levels of exogenous NO added in the experiments from [26]) in order to establish baseline responses of the epi-cell agent's TJ protein synthesis/localization algorithms and link them to the permeability data seen in the corresponding bars in Figure 5. The Cytomix bars in this Figure 4 are used to calibrate the iNOS-NO production algorithms within the epi-cell agents. The middle data set (grey bars = Cytomix + NO scavenger) show the effect of exogenous NO reduction/elimination on the generated levels of NO in the face of Cytomix. This graph can be compared to the upper panel of Figure 1 in Ref [26].

**Figure 7:**
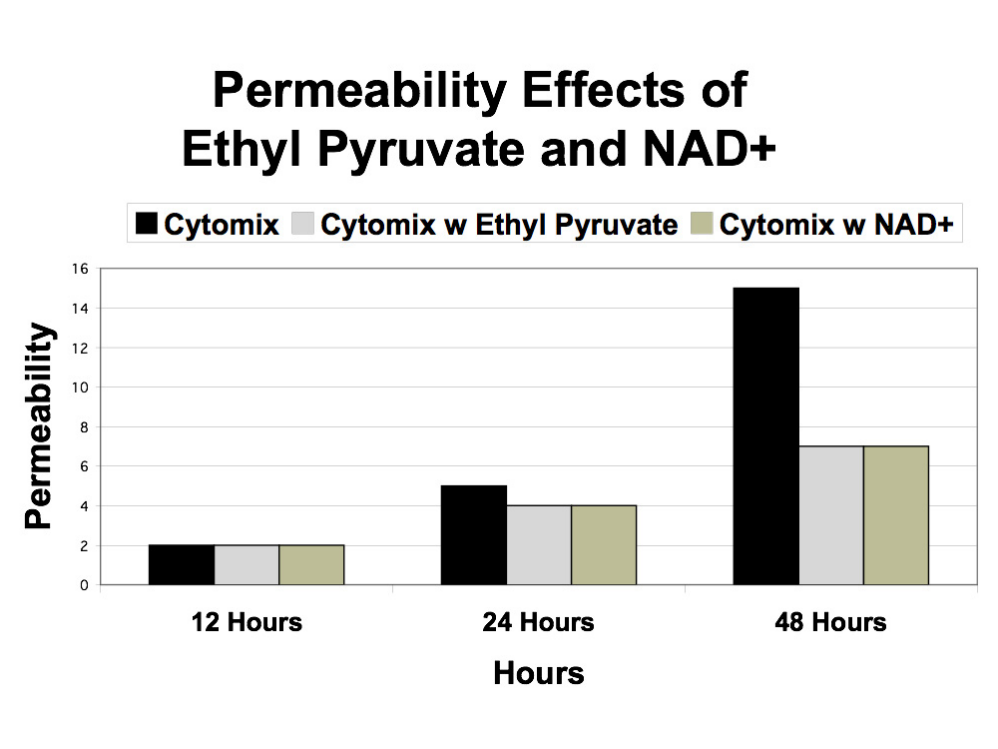
**Simulated Permeability Effects of Ethyl Pyruvate and NAD^+^**. Graph demonstrating the effects of simulated addition of ethyl pyruvate and NAD^+ ^on the pro-inflammatory algorithms within the epi-cell agents. Both of these substances interfere with NF-kappa-B localization, and therefore are "upstream" from the iNOS-NO pathways as represented in those rules. This graph can be compared to Figure 1 from Ref [24] with ethyl pyruvate at 1.0 mM dose, and Figure 1a from Ref [25] with NAD^+ ^at 0.1 mM dose.

**Figure 8:**
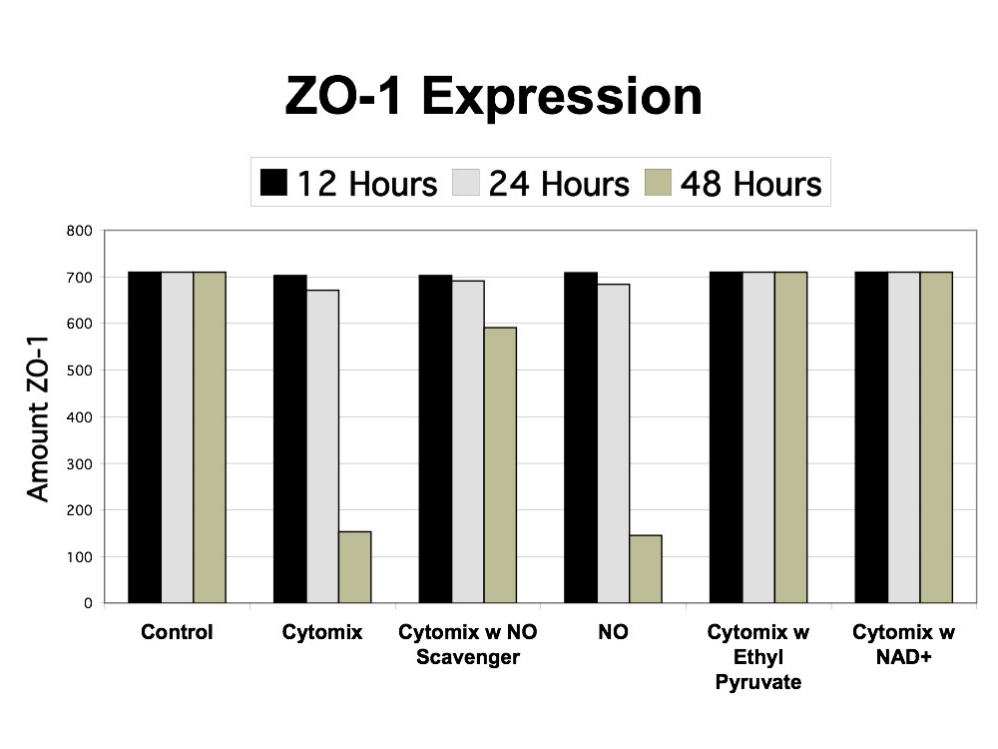
**Simulated Levels of ZO-1 Expression**. Graph demonstrating the levels of simulated ZO-1 expression in control, exogenous NO, Cytomix, Cytomix with NO scavenger, Cytomix with ethyl pyruvate and Cytomix with NAD^+ ^at 12 h, 24 h and 48 h. Compare with Figure 6 from Ref [24] and Figure 2 from Ref [25] (latter is extrapolated from Western blot analysis).

**Figure 9:**
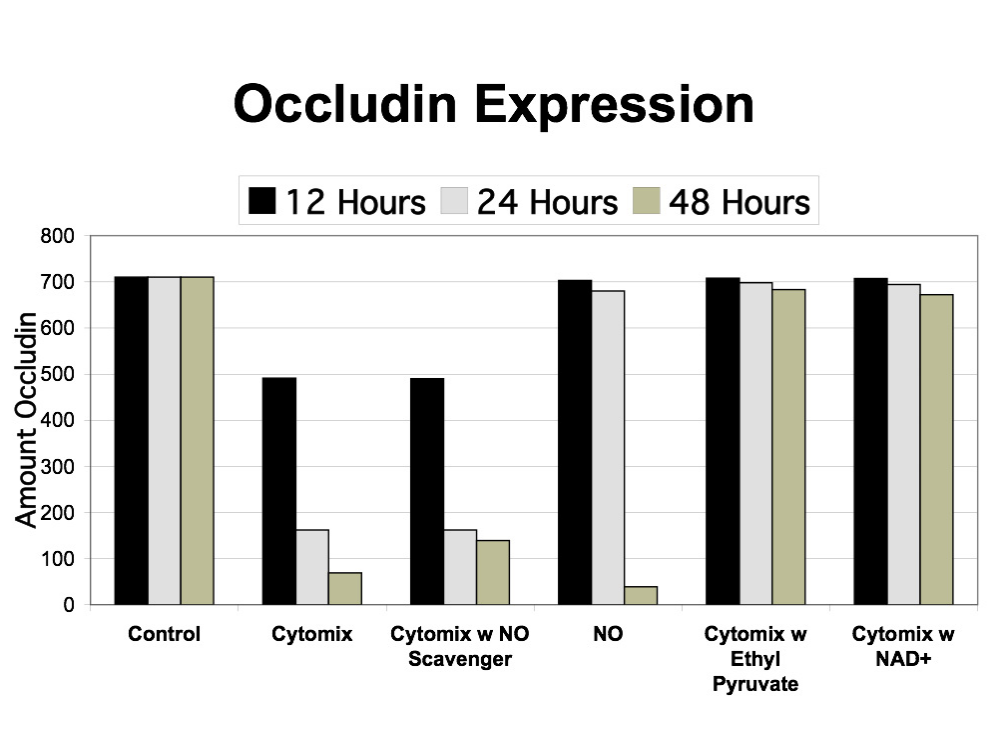
**Simulated Level of Occludin Expression**. Graph demonstrating the levels of simulated occludin expression in control, exogenous NO, Cytomix, Cytomix with NO scavenger, Cytomix with ethyl pyruvate and Cytomix with NAD^+ ^at 12 h, 24 h and 48 h. Compare with Figure 6 from Ref [24] and Figure 2 from Ref [25] (latter is extrapolated from Western blot analysis).

The results of the in-silico runs of the EBABM can be seen in Figures 5, 6, 7, 8 and 9. Note that the values of the in-silico experiments are unit-less, but the results qualitatively mirror the reference data set. Calibration results can be seen in Figures 5 and 6. Both of these figures include runs with exogenous NO, cytomix and cytomix in the presence of a NO scavenger. Figure 5 demonstrates the calibrated levels of NO production, while Figure 6 demonstrates the permeability calibration results. These figures essentially reproduce the data generated in Ref [26].

The effects of the interventions represent the validation step in the evaluation of the EBABM. Figure 7 demonstrates the effects of ethyl pyruvate and NAD^+ ^on permeability, with the data in Figure 6 representing the control arm. The reference data for the effect of these interventions on the permeability changes with cytomix administration can be seen in Figure 1 from Ref [24] with ethyl pyruvate at 1.0 mM dose, and Figure 1a from Ref [25] with NAD^+ ^at 0.1 mM dose. Figures 8 and 9 reproduce the results seen extrapolated from the Western Blot data on the effect of ethyl pyruvate and NAD^+ ^administration on TJ proteins, specifically ZO-1 and occludin (Figure 6 from Ref [24] and Figure 2 from Ref [25]). ZO-1 is significantly decreased at 48 hours, while occludin starts to drop at 24 hrs with the cytomix and continues to decrease at 48 hrs, but has a profile more similar to ZO-1 when run with the exogenous NO only. The simulation of adding both ethyl pyruvate and NAD^+ ^both obviated the effects of both exogenous NO and cytomix on both ZO-1 and occludin.

### Development of the organ level ABMs

**Figure 10:**
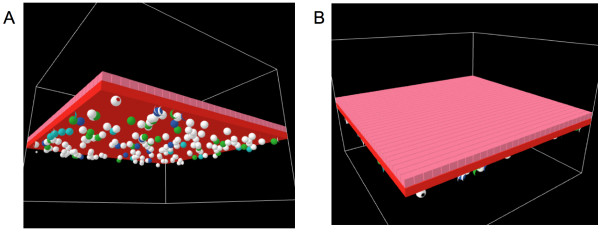
**Screenshots of Bilayer Gut ABM**. Bilayer configuration of the gut ABM, following the structure for "hollow" organs described in the text. Figure 10a is the view of bilayer from endothelial surface. Red cubes represent endothelial cell agents, with spherical inflammatory cell agents seen just below. Inflammatory cell agents move in the plane immediately below the endothelial surface, and these interaction rules are derived from the Innate Immune response ABM from Ref. [6]. Figure 10b is the view of bilayer from epithelial surface. Pink cubes represent epithelial cell agent, governed by rules transferred from the EBABM. Impairment of TJ protein metabolism is shown by darkening of the color of the epithelial cell agent, with the epithelial cell agents eventually turning black and changing their shape to a "cone" when TJs have failed (see Figures 11, 14–16).

As discussed above, the next level of ABM development is intended to simulate organs as a concatenation of two distinct hypotheses of disseminated inflammation and organ failure: that of endothelial dysfunction and that of epithelial dysfunction. Therefore the structure of these models involves the 3-dimensional linkage of the cellular surface ABMs already developed representing these two systems. The result is a "bilayer" organ model (see Figures 10). With this abstraction many organ systems can be functionally and morphologically represented. "Hollow" or "luminal" organs are those that present an epithelialized surface to the external environment, while retaining an "internal" intercommunication surface via a blood capillary interface. Examples of such organ systems would be the lungs, the gut, the kidney, the liver and (topologically) the skin. While there would obviously be differences between the functions of the various epithelial cells depending upon their organ of residence, to a great degree the central goal of maintaining the "integrity of self" is done through sustaining epithelial barrier function via the ubiquitous mechanism of tight-junction integrity [19].

### Reference model for the organ ABM: in vivo models of gut ischemia and inflammation

In vivo models that examine the inflammatory behavior of the gut either look at a local effect from direct occlusion of gut arterial flow [21,32,33] or as a result of some systemic insult, be it hemorrhagic shock [34-36], endotoxin administration [37,38] or burn injury [39,40]. These studies suggest that the primary process that initiates inflammation in the gut is ischemia and reperfusion, and the subsequent effects on the endothelial surfaces within the gut. The measurable outputs of the reference models exist at different scales. At the cellular level, tight junction integrity and epithelial barrier function is one measured endpoint [41,42], however the organ as a whole also has an output: the nature of the mesenteric lymph. Multiple studies suggest that ischemia to the gut (and subsequent inflammation) leads to the excretion of an as-of-yet unidentified substance in the mesenteric lymph that has pro-inflammatory qualities. Some characteristics of the substance can be identified from the literature: it is an acellular, aqueous substance [43], is greater than 100 kD in size [44], does not correspond to any currently recognized cytokine, and is bound or inactivated by albumin [45]. The time course of the production of the substance is identified to some degree [35,46] but it is unclear if it arises from a late production of inflamed cells, or is a product of cellular degeneration or apoptosis, or is a transudated bacterial product from the intestinal lumen. The uncertainty with respect to an identified mediator provides a good example of how the ABM architecture deals with incomplete knowledge. Based on the characteristics defined above, we make a ***hypothesis ***regarding this substance with respect to its origin, but acknowledge that this is, to a great degree, a "best guess." Doing so establishes a "knowledge bifurcation point," allowing the development of potential experiments and/or data that would "nullify" the particular hypotheses. A specific example will be demonstrated below.

### Organ ABM: construction

Both the original endothelial/inflammatory cell ABM and the EBABM were developed as 2-dimensional models. In order to create the bilayer topology of the organ ABM it was necessary to convert both of these models to the 3-dimensional version of Netlogo, with each model represented as a layer of agents projected in the XY plane. The two layers were then juxtaposed, the endothelial layer below and the epithelial layer above along the Z-axis. The simulated blood vessel luminal space occupied another XY plane one place inferior to the endothelial surface along the Z-axis. Inflammatory cells move only in this plane. The organ luminal space occupied the XY plane at one place superior to the epithelial axis along the Z-axis. This space contains the "diffusate" that leaks into the gut in cases of epithelial tight junction failure. For a screenshots demonstrating the topology of this model see Figures 10.

The nature of the initial perturbation was altered to match that seen in the reference experiments, i.e. tissue ischemia. With the premise that the inflammatory response was generated at the endothelial surface the initial perturbation was modeled focusing at the endothelial layer, with the response of the epithelial component being subsequently driven by the output of the endothelial-inflammatory cell interactions. Rather than having a localized insult with either infectious agents (simulating infection) or sterile endothelial damage (simulating tissue trauma) as was the case in the base endothelial/inflammatory cell ABM, gut ischemia was modeled as a percentage of the total endothelial surface rendered "ischemic," a state defined in the rules for the endothelial cell agents as an "oxy" level < 60. The affected endothelial cell agents were randomly distributed across the endothelial surface. The degree (or percentage affected) of the initial "ischemia" was controlled with a slider in the Netlogo interface. Therefore "Percentage Gut ischemia" (= "%Isch") represents the independent variable as initial perturbation for this model. Other than the changes noted above, no other changes to the rules of either the endothelial/inflammatory ABM or the EBABM were made.

To address the issue of modeling the production of post-ischemic, pro-inflammatory lymph, attention is focused on linking the knowledge that has been acquired regarding the characteristics of the substance, and relating this information to the components of the organ ABM. The known characteristics listed above are used to exclude potential candidate-substances/actors from consideration. Specifically, this group comprises any of the cellular agents and any of the included cytokines. Therefore, the search is limited to:

1) An as-of-yet unidentified compound linked to cellular damage. An example of such a compound would be high-mobility box protein 1 (HMGB-1), which to date has not been looked for in post-ischemic mesenteric lymph. In the organ ABM this variable is termed "cell-damage-byproduct," and it is calculated as a function of total endothelial damage with a set decay rate consistent with that of other bioactive compounds associated with inflammation.

2) A luminal compound that diffuses in response to TJ barrier failure. This would correspond to potential byproducts of gut bacterial metabolism, or bacterial toxins, or other soluble aspects of the gut luminal environment that would leak into the gut tissue by virtue of the loss of barrier function. This variable is represented by "gut-leak," which is equal to the "solute" (from the EBABM) that penetrates the failed barrier.

3) A down-stream metabolite of compounds generated by the inflammatory process. These would most likely be compounds generated by superoxide and NO reactions. For purposes of these simulations, levels of NO will be used as a proxy for this possible candidate.

Therefore, the goal of the organ ABM simulation runs will be to examine the time course levels of these three values and identify which one (if any) matches the reported time course effects of the post-ischemic mesenteric lymph.

### Organ ABM: simulations and results

The initial goal with the organ ABM simulations was to determine the greatest non-lethal level for "Percentage Ischemia" (%Isch). It should be noted again that the name of this variable is descriptive for how it is implemented in the ABM, and not intended to match quantitatively, per se, with measured ischemia in vivo. Rather "%Isch" is representative of the initial conditions for the simulation that will produce a pattern of simulation behavior that matches that of the in vivo system [20]. A parameter sweep of this value was performed, using a previously described method [5] with the goal of identifying the greatest non-lethal level for %Isch. This value was determined to be 35, and will be used as the initial condition for the subsequent organ ABM runs.

**Figure 11:**
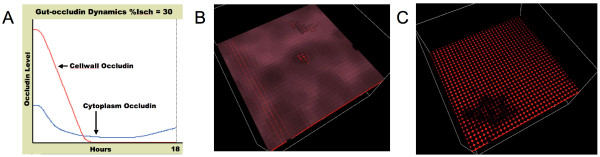
**Gut ABM with %Isch = 35 at 4 and 18 h**. Figure 11a is a graph that demonstrates the timecourses of Cellwall Occludin and Cytoplasmic Occludin over 18 hrs with an initial "%Isch" = 35. The Cellwall Occludin is shown in Red, the Cytoplasm Occludin is shown in Blue. Note that Cytoplasm Occludin has started to recover at ~12 h. The delay in recovery of the Cellwall Occludin is due to the persistent effect of NO on Occludin localization via the NSF pathway. Figure 11b is the view of gut ABM from the epithelial surface at 4 hours. Note the darkened sections of the epithelial surface, denoting impaired TJ protein metabolism and localization. The areas near the center and right upper corner of the layer show the change in shape from "cube" to "cone," indicative of TJ failure. Figure 11c is the view of gut ABM from the epithelial surface at 18 hours. Note how there has been progression to generalized TJ barrier failure, with only the small area in the left lower corner still with some intact TJs.

The initial experiments with the organ ABM examined the effect of gut ischemia on TJ protein metabolism and the consequent effect on epithelial barrier function. The primary purpose of these experiments was to confirm that the epithelial agents' TJ metabolism and inflammatory signaling rules, as transferred from the EBABM, retained time course validity when the initiating stimuli were generated from endothelial/inflammatory cell interaction instead of exogenously administered cytomix. Figure 11 demonstrate the behavior of the gut ABM with "%Isch" = 35. In Fig 11a occludin levels (as a representative TJ protein) decreased during an 18 h period post insult, and barrier function can be seen disturbed at 4 and 18 h (Figs 11b and 11c), consistent with that seen in the published literature [41,42].

**Figure 12:**
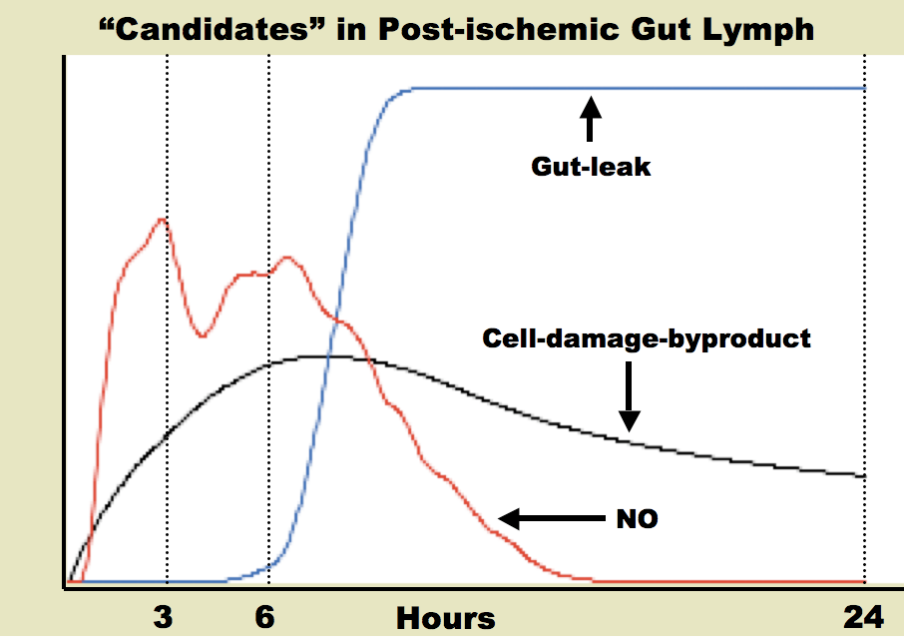
**Timecourses for "Candidate" variables in Post-ischemic Gut Lymph**. Graph of the dynamics of three potential "candidates" for the yet unidentified pro-inflammatory compound seen in post-ischemic mesenteric lymph. Note that the units of the three graphs have been adjusted to show their time-courses side-by-side; the emphasis is on the patterns of the timecourses rather than the absolute values. The "cell-damage-byproduct" graph best follows the reported characteristics of post-ischemic mesenteric lymph, with the greatest rises in pro-inflammatory activity at 3 and 6 h, and persisting to 24 h. "NO" rises early enough for the pro-inflammatory effect at 3 and 6 h, but is not present at 24 h. The "Gut-leak" is delayed in its rise, and therefore cannot account for activity seen at 3 and 6 h.

The output from a representative run of the organ ABM with %Isch = 35 is shown in Figure 12, where the time courses for "cell-damage-byproduct" (black line), "gut-leak" (blue line) and NO (red line) can be seen. The pro-inflammatory properties of the post-ischemic mesenteric lymph are noted to increase the most at 3 h and 6 h and remain out to 24 h [35,46]. Examining the time courses shown in Figure 12, the candidate compound that most closely approximates the pattern identified in the literature is the "cell-damage-byproduct." As discussed above, this possible source of the unknown compound in post-ischemic mesenteric lymph is based on the recognition of certain "late" pro-inflammatory mediators produced by activated and damaged cells, HMGB-1 being the most studied as a possible key mediator in the pathogenesis of sepsis [47]. To date, there have been no studies examining the production or presence of HMGB-1 in post-ischemic mesenteric lymph. However, based on the information generated by the organ ABM, and placed in the context of the knowledge framework concerning the characteristics of pro-inflammatory mesenteric lymph, we will make a ***hypothesis ***that some "later" byproduct of damaged gut tissue, rather than a diffused material or direct metabolite of first-pass inflammatory mediators, is the responsible compound in post-ischemic mesenteric lymph. It is recognized that this is "guided speculation;" however, it also demonstrates how the construction and use of models in the ABM architecture is an evolving process that parallels the development and refinement of conceptual models. As will be seen in the next section, the next scale of biological organization to be addressed in the ABM architecture involves the extension and integration of this hypothesis.

### Development of multi-organ ABM: the gut-pulmonary axis of inflammation

Organs do not exist in isolation; their mutually complementary functions interact to sustain the organism as a whole. Unfortunately, disease states can lead to a breakdown of these interactions, causing a cascade effect as single organ dysfunction can lead to multiple system failure. Sepsis and MOF are characterized by a progressive breakdown in these interactions, leading to recognizable patterns of linked organ failure [48]. Therefore the next scale of biological organization represented in the multi-scale ABM architecture is that of organ-organ interaction. The gut-pulmonary axis of multiple organ failure [22,36,40,46] is used as the initial example of organ-to-organ crosstalk. This relationship is relatively well defined pathophysiologically (though not completely, as indicated by the uncertainty of the identity of the pro-inflammatory compound in post-ischemic mesenteric lymph) and represents an example of multi-organ effects of disseminated disordered inflammation. Disordered acute inflammation of the lung is termed Acute Respiratory Distress Syndrome (ARDS), and is manifested primarily by impaired endothelial and epithelial barrier function, leading to pulmonary edema. This leads to impaired oxygenation of arterial blood, requiring support of the patient with mechanical ventilation. While the comprehensive pathogenesis of ARDS involves additional subsequent issues related, to a great degree, to the consequences of mechanical ventilation (specifically the effects of barotrauma and shear forces on the airways, and the persistent propagation of inflammation that results), for purposes of this initial demonstration only the initiating events associated with the development of ARDS will be modeled. Those events concern the production and release into the mesenteric lymph by ischemic gut (resulting from shock) of various pro-inflammatory mediators, and their effects both on circulating inflammatory cells and the pulmonary endothelium as they circulate back to the lung via the mesenteric lymph (as discussed above)[21,22,36,40,49,50]. At this point, the hypothesis regarding the nature of the pro-inflammatory mediator in the mesenteric lymph is extended to the assumption that, for modeling purposes, the levels of "cell-damage-byproduct" will be the proxy for the unidentified compound that is produced in the ischemic gut and circulated to the lung, leading to inflammation of pulmonary endothelium.

### Extension of gut ABM to pulmonary ABM

Thus far emphasis has been on the development of the gut organ ABM, and in order to model gut-pulmonary interactions it is necessary to develop a pulmonary ABM as well. Drawing upon the endothelial-epithelial bilayer configuration for a "hollow" organ, the pulmonary ABM utilizes the same endothelial-inflammatory cell component as the gut ABM, predicated on the relative homogeneity in structure and function of capillary endothelial cells (the blood brain barrier being the notable exception). Furthermore, pulmonary epithelial cells behave very similarly to gut epithelial cells with respect to tight junction metabolism and epithelial barrier function [51]. Therefore the pulmonary epithelial agent layer also utilizes the same rules as the gut ABM epithelial agents with respect to these processes. There is, however, a difference in function of the intact epithelial barrier, and the consequence of its failure. The functional consequence of the intact pulmonary epithelial barrier is effective oxygenation of arterial blood (expressed at the endothelial lumen) via diffusion from the alveolar epithelial surface. Pulmonary barrier failure manifests as increased egress of fluid from the endothelial lumen into the alveolar space. The effect of pulmonary diffusate "leak" is modeled to affect the transfer of alveolar oxygen to the endothelial surface. Thus far the "oxy" level in both the base endothelial-inflammatory cell ABM and the gut ABM is set at 100 for all non-perturbed endothelial cells, predicated upon the assumption of constant adequate pulmonary function. Now, with the modeling of inflammation that would affect the efficacy of systemic oxygenation (i.e. the lung), the systemic oxygenation may be altered with the consequence that progressive pulmonary dysfunction would feed back to the system as whole. Thus the influence of the pulmonary inflammation with respect to decreased pulmonary epithelial barrier function, leading to increased diffusate "leak" into the alveolar space. This in turn leads to impaired oxygenation into the endothelial lumen, which is summed across the surface of the model to produce a measure of systemic arterial oxygen content. This value will now represent the baseline "oxy" level for all other systemic endothelial agents.

### Multi-organ ABM: construction

**Figure 13:**
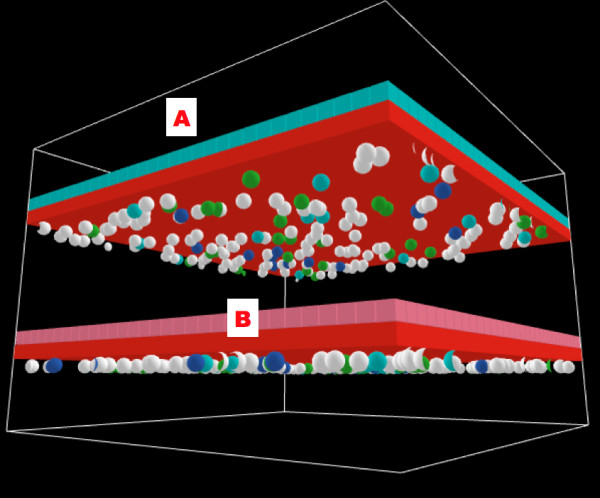
**Screenshot of Multi-Bilayer Gut-Lung Axis ABM**. The multiple bilayer topology of the Gut-Lung ABM is seen here. Letter A labels the pulmonary bilayer, with Aqua cubes representing pulmonary epithelial cell agents, Red cubes representing pulmonary endothelial cell agents, and below are spherical inflammatory cell agents. Letter B labels the gut bilayer, with a similar configuration, the only difference being that gut epithelial cell agents are Pink. Circulating inflammatory cell agents move between these two bilayers in the fasion described in the text.

Following the development of both a gut ABM and a pulmonary ABM, the next step is to connect them in a linked model. The topology of this relationship consists of two parallel bilayer planes, each bilayer representing one of the organ ABMs (Figure 13). This is the gut-lung-axis ABM. The Z-axis orientation of both bilayers is the same, to allow conservation of the agent rules for equivalent agent classes (i.e. endothelial-epithelial-lumen relationships are consistent). The simulated blood flow continues to be modeled by movement in the XY plane immediately inferior to the endothelial surface. Blood flow between organs is simulated by adding a "perfusion" variable. "Perfusion" refers to the time-steps that a circulating cell remains in one organ bed before being transferred to the other organ bed. For purposes of the model, large caliber blood vessels and the heart are treated as biologically inert with respect to inflammation. Perfusion time in each organ is simulated at approximately 6 minutes, accounting for the general slowing of cellular flow during movement through the capillaries and venules. The activation of adhesion molecules on the circulating cells and corresponding endothelial cells leads to increased time in an organ bed, or, in the case of adhering and migrating cells, persistence in one organ bed. On leaving the organ bed, movement from one capillary bed to the other is assumed to take less than one time step (<3 min) as cells are transferred directly to a random position on the other organ's endothelial surface at the end of their "perfusion" interval. Similarly, the flow of mesenteric lymph is modeled with a new command "gut-lung-lymph-flow" in which the level of "cell-damage-byproduct" is transferred from the gut ABM endothelial space to the lung ABM endothelial space. There is no equivalent flow of "cell-damage-byproduct" in the other direction, though the effect of the lung ABM on baseline systemic oxygenation (as described above) represents the connection in the direction from the lung to the gut. The endothelial activating properties of "cell-damage-byproduct" are modeled by having this variable activate its adjacent pulmonary endothelial agent in a manner similar to "endotoxin," i.e. increasing levels of "endo-selectin" and "endo-integren" levels, and producing platelet activating factor ("PAF") and "IL-8".

### Multi-organ ABM: simulated interventions and results

**Figure 14:**
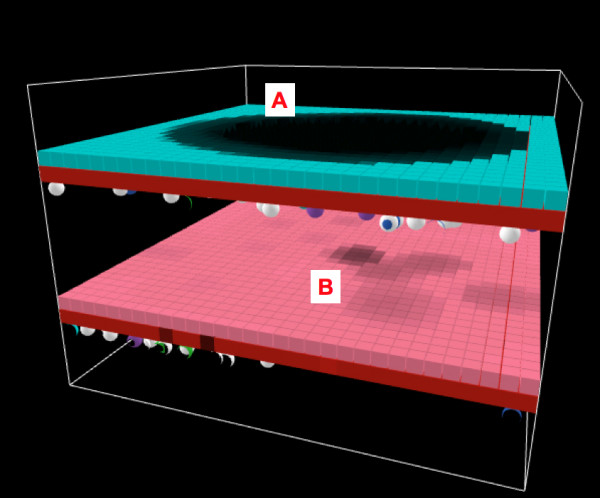
**Simulated "Pneumonia" with effect on Gut Barrier Dysfunction**. This panel shows a representative run of the Gut-Lung Axis ABM with "pneumonia" as the initial perturbation. Letter A demonstrates the localized injury to the pulmonary bilayer, Letter B demonstrates areas of the gut epithelial layer that are starting to have impaired TJ protein metabolism due to gut ischemia from decreased systemic oxygenation arising from pulmonary leak.

Two clinical conditions were simulated to model organ-organ crosstalk along the gut-pulmonary axis of inflammation. The first has already been discussed at length: gut ischemia resulting from shock, demonstrating the effect that the gut has on the lung. The second will be a primary pulmonary process that will in turn affect the gut: pneumonia. "Pneumonia" is modeled as in infectious insult to the lung ABM using the same initial infectious insult rules as for the base endothelial-inflammatory ABM [5,6]. Infectious agents are added in a localized fashion, and damage endothelial cells, produce "endotoxin" when killed and replicate if not suppressed. In the gut-lung-axis ABM the resulting damage to the lung and increased simulated pulmonary edema impair systemic oxygenation, and this leads to gut ischemia. Also, activation of inflammatory cell agents by the infectious agents potentiates their pro-inflammatory behavior once they are in the gut ABM endothelial surface. An example of simulated "pneumonia" is seen in Figure 14. Again, it should be noted that the simulated conditions and interventions at the organ level are admittedly abstract; it is not the intent of this demonstration to simulate the complete complex pathophysiology of pneumonia and sepsis. However, the aspects of both pneumonia and sepsis that are simulated do represent the central processes involved, and serve to illustrate the capability of this modeling architecture to represent the interactions, based on what is recognized in the literature, between these two organ systems.

**Figure 15:**
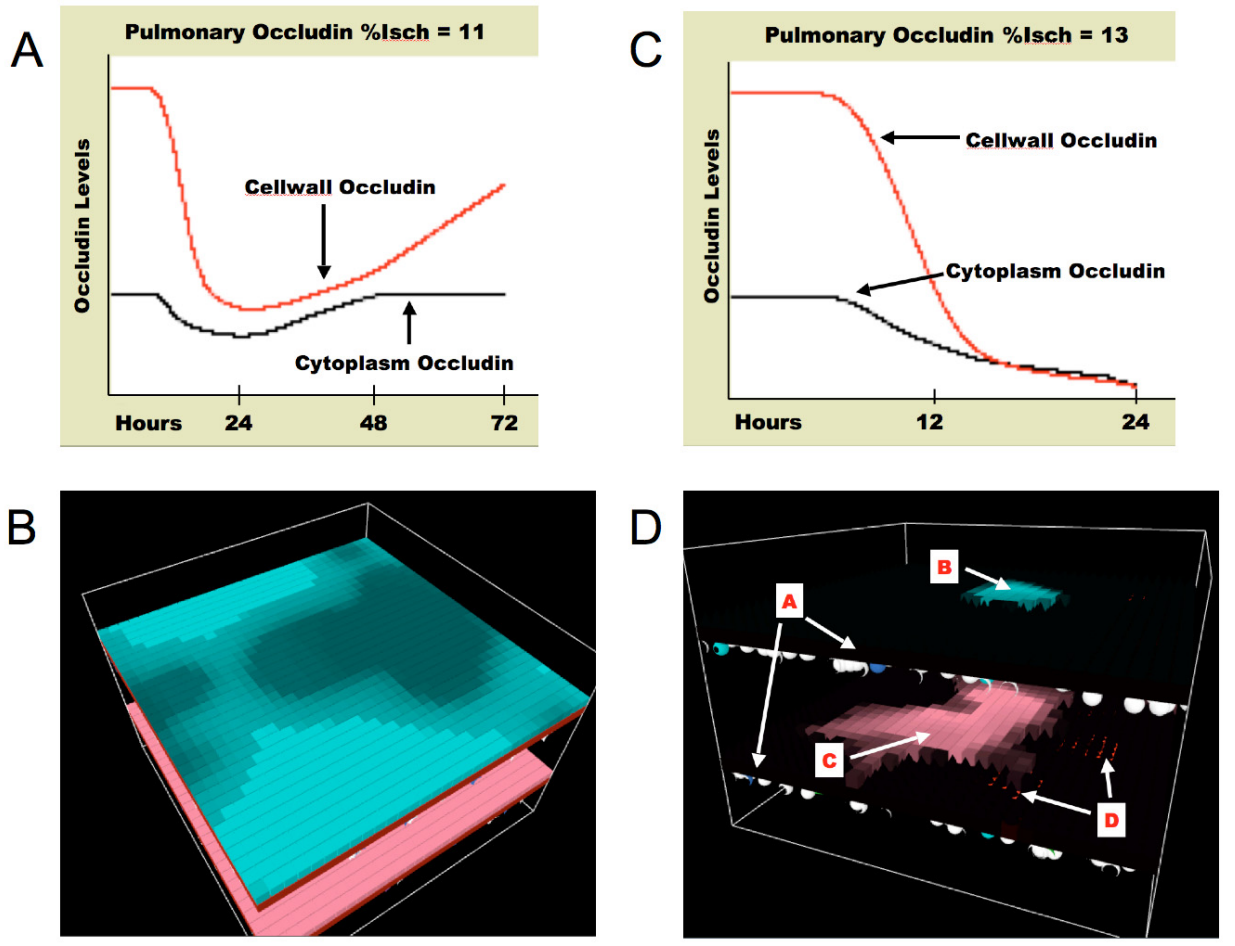
**Effect of Gut Ischemia on Pulmonary Barrier Dysfunction and Pulmonary Edema**. Figure 15a shows the dynamics of pulmonary occludin levels (as a proxy for pulmonary barrier dysfunction) in a representative run with a sub-lethal initial "%Isch" = 11 over a 72 hour run. Levels of both Cytoplasm Occludin and Cellwall Occludin nadir at ~24 hrs, then show gradual recovery as inflammation subsides. TJ protein levels continue to rise towards 72 hours. This pattern is consistent with that seen clinically in the recovery of pulmonary edema secondary to inflammatory causes. Figure 15b shows a screenshot for this representative run at the end of 72 hours, demonstrating a mostly recovered pulmonary epithelial surface. Figure 15c shows the dynamics of pulmonary occludin levels in a representative run with a lethal initial "%Isch" = 13. Level of both Cytoplasm Occludin and Cellwall Occludin are seen to drop consistent with progressive activation of pulmonary endothelium and production of NO, leading to pulmonary TJ failure. This run is terminated at 24 because endothelial damage is nearly complete, as seen in the corresponding screenshot in Figure 15d. Figure 15d Letter A points to black cubes representing "dead" endothelial cell agents. These agents "die" owing to a decrease in the available maximal "oxy" level to below the threshold for generalized endothelial agent activation. The impaired systemic oxygenation due to pulmonary leak arises from pulmonary epithelial barrier failure. Letter B points to the only remaining intact pulmonary epithelial cell agents. Letter C points to the only remaining intact gut epithelial cell agents. Letter D points to the only remaining patches of surviving endothelial agents (red areas seen through the failed gut barrier).

Figure 15 demonstrate the effects of mesenteric ischemia on pulmonary barrier dysfunction. Note that the sub-lethal "%Isch" has been dropped to 11 (Figure 15a and 15b), while the "%Isch" = 13 results in a lethal dynamic (Figures 15c and 15d). As expected, the corresponding lethality of mesenteric ischemia in the gut-lung ABM is significantly increased as compared to the gut ABM alone, dropping the sub-lethal "%Isch" from 35 for the gut ABM to 11 for the gut-lung ABM. This results from the addition of the lung ABM and its effect of decreasing the maximally available "oxy" to non-perturbed endothelial agents via the consequence of pulmonary epithelial barrier function ("pulm-edema"). The "survival space" of the system is therefore greatly limited, and it may initially appear that this model would be unsuited to examining the range of dynamics of interest in the study of sepsis. However, it should be noted that the high lethality of mesenteric ischemia, which implies the presence of hemodynamic shock, is "historically" correct. Shock states, prior to the development of fluid resuscitation and respiratory support, were nearly universally fatal. This is the circumstance that is being represented with the gut-lung ABM at this point. If the goal is to simulate the clinical conditions associated with sepsis and MOF, then it is necessary to simulate the effects of organ support, to shift the "survival space" to the right. Doing so reproduces the fact that sepsis and MOF are a "disease of the ICU," arising only after the advances of resuscitative, surgical, antimicrobial and organ-supportive care allowed the maintenance of patients in situations where they previously would have died. Therefore, sepsis and MOF can be thought of as a previously unexplored behavior space of systemic inflammation, one where the inflammatory system is functioning beyond its evolutionarily defined design parameters [5,6].

**Figure 16:**
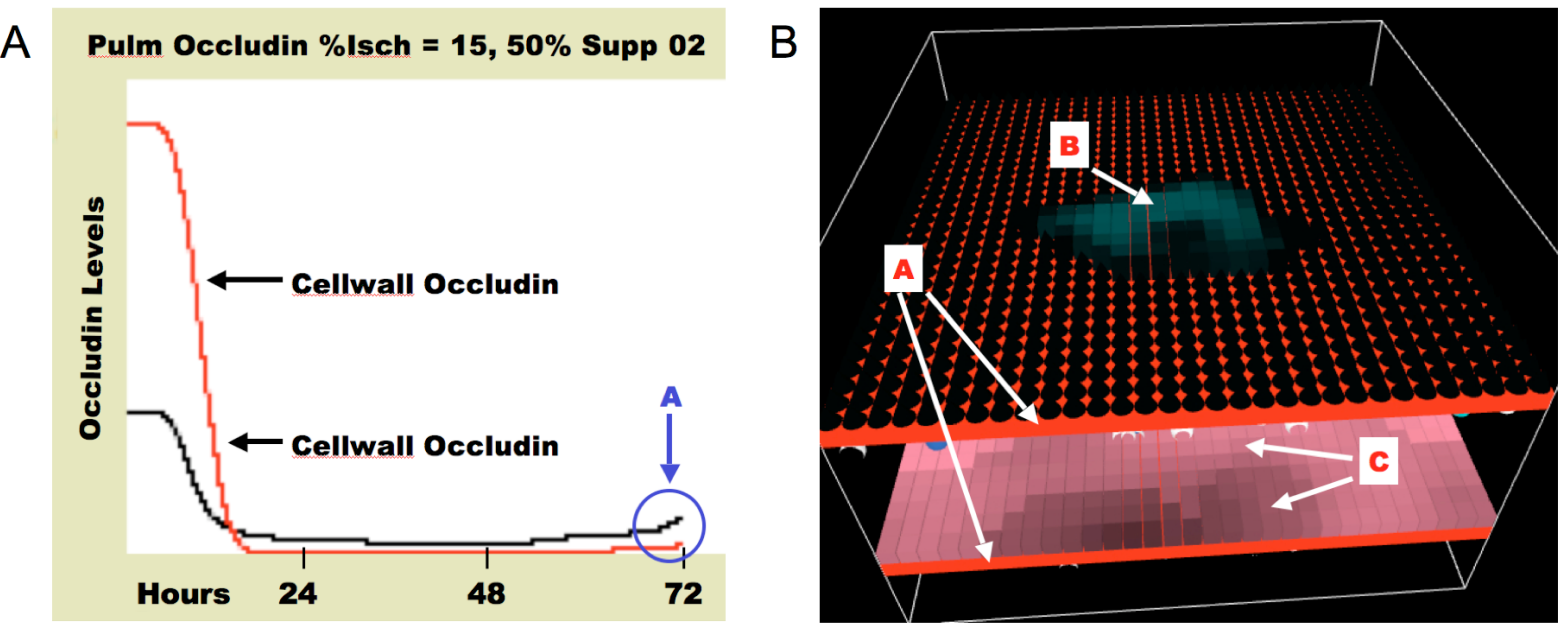
**Effect of Simulated Supplementary Oxygen on dynamics of simulated Pneumonia**. Figure 16a demonstrates the dynamics of pulmonary Cytoplasm and Cellwall occludin in a representative run with an initial "%Isch" = 15, and the addition of simulated organ support in the form of "Supplementary Oxygen" at 50%. The effect of "Supplementary Oxygen" is additive to the level of "oxy" generated by the lung ABM and distributed to the endothelial surface. The initial drop of the pulmonary Occludins is consistent with inflammatory effects of post-ischemic mesenteric lymph. The effect of the "Supplementary Oxygen" is to blunt the effect of the resulting pulmonary edema, and it keeps the "oxy" level above the threshold ischemic level for activation of the generalized endothelial cell agent population. As a result the endothelial surface if maintained through the period of most intense inflammation, and allows the epithelial cells to begin recovery of their TJs (see Letter A in Figure 16a). The stabilization and initiation of recovery of pulmonary epithelial TJs at 72 hours is consistent with the clinical time course of adult respiratory distress syndrome due to an episode of shock. Figure 16b is a concurrent screenshot of the representative run. Letter A demonstrates the intact endothelial agent layer due to "Supplementary Oxygen" support (compare with Figure 15c, Letter A). Letter B demonstrates the recovering pulmonary epithelial cell agents. Letter C demonstrates intact and recovering gut epithelial.

Therefore, to accomplish this goal, a very abstract means of organ support is modeled in the form of "supplementary oxygen." This function increases the amount of "oxy" that is able to be diffused through the pulmonary epithelial barrier and therefore available as systemic oxygenation. This is the qualitative equivalent of increasing the fraction of inspired oxygen, and therefore alveolar oxygen, and therefore can increase the partial pressure of oxygen diffused in the blood. It is qualitative in so much as there is no attempt to reproduce the dynamics of gas exchange, or the binding of hemoglobin to oxygen in the blood, or the effects of redistributed ventilation-perfusion matching in the lung as a result of hypoxia. This degree of detail is beyond the scope of this initial demonstration model; however the qualitative behavioral effects do show that this type of support, even abstractly modeled, increases the richness of the behavior of the model as a whole, and can extend the examinable behavior space of the model to situations that can approximate the effects of organ support in the ICU. The corresponding changes in outcome with this type of simulated organ support can be seen in Figure 16.

This sequence illustrates an important point in creating translational models of disease states. The tendency may be to attempt to model the pathological state being studied, i.e. creating a model of sepsis. However, it needs to be remembered that pathological states result from transitions from normal physiological behavior, and if the intent of a model is to facilitate the eventual transition from disease back to health, then "normal" mechanism must be the basis of a translational model. The need to capture transitions from one state to another takes on further importance when the pathological state results, as with sepsis, from medical/clinical interventions. Therefore, the architecture of a modeling structure needs to be flexible enough to accommodate the addition and integration of these factors, and it is hoped that the presented modular structure of the ABM architecture demonstrates this capability.

## Discussion

The biomedical research community today faces a challenge that has paradoxically arisen from its own success: as greater amounts of information become available at increasingly finer levels of biological mechanism it becomes progressively more difficult for individual researchers to survey and integrate information effectively, even within their own area of expertise. Though technology, via tools like PubMED, the introduction of new publication formats like open-access journals and the development of a whole slew of bioinformatics tools, has aided the distribution and availability of biomedical information, it still falls upon the individual researcher to concatenate that information into a conceptual model that represents it. These mental models guide the direction of their individual research and, in aggregate, the form the components of the evolving structure of community knowledge. However, the formal expression of mental models remains poorly defined, leading to limitations in the ability to share, critique and evolve the knowledge represented in these conceptual models, particularly across disciplines. As a result it is increasingly difficult for both the individual researcher, and the community as a whole, to "know what it knows."

These limitations can be overcome by developing methods of formal dynamic knowledge representation to allow researchers to express and communicate their mental models more effectively. Furthermore, in order to be able to "see" the consequences of a particular hypothesis-structure/mental model, the formally represented knowledge should be moved from a static depiction to a dynamic model in which the mechanistic consequences of each hypothesis can be observed and evaluated. In addition, as seen in the example of modeling the pro-inflammatory aspects of the post-ischemic mesenteric lymph, a method of formal dynamic knowledge representation also allows researchers to propose alternative solutions and generate hypotheses in the process of creating a model, *so long as these hypotheses and assumptions are made explicit*. This is necessary in any attempt to formalize the representation of conceptual models, as it will always be necessary to deal with the issue of incomplete knowledge. These models can aid in the scientific process by providing a transparent framework for this type of speculation, which can then be used as "jumping off" points for the planning and design of further wet lab experiments and measurements. I propose that ABM is a method well suited to fulfilling the goals of dynamic knowledge representation.

This paper has presented a series of ABMs that are intended to introduce a multi-scale architecture that has the potential to serve as an overall unifying structure for representing biomedical knowledge. The "encapsulation" represented by the agent-based paradigm does not preclude the development of equation based or stochastic models; rather the modular, encapsulating structure is agnostic to the nature of the agent rule systems, and agnostic to the method of linkage to the various components. This is consistent with the "functional unit representation method" (FURM) concept developed by Hunt [52,53]) in which computational models of biological systems would be assemblies of methodologically agnostic components. To state this in multi-scalar terms, such a architecture would allow each level of organization to be modeled with a methodology, or multiple methodologies, most suited to its particular characteristics [54,55]). As a result there is an expectation that these assembled-models would be hybrids of different modeling techniques [2,14]).

The ABMs presented herein represent admittedly abstract representations of mechanistic hypotheses, but this need not be the case. Equations "encapsulate" knowledge as well, by providing mathematical abstractions of behavior that none-the-less must actually be implemented by some biological object. The extensive work on the mathematical characterization of intracellular processes in the systems biology field can form the basis of cell-level agent rules. In particular, the encapsulation offered by the ABM paradigm offers a method of meeting current challenges in the application of mathematical modeling techniques, such as parallel implementation of stochastic modeling with Gillespie algorithms, to reproduce population behavior and transcend biological scales of organization. The complexity and detail of these models is constrained only by the scope of that knowledge, and the ability to compute subsequently expressed rules. The former is the subject of the ongoing scientific process aimed at identifying mechanisms; the latter is the being addressed by a concurrent research community that seems to follow, at worst, the exponential progress represented by Moore's Law.

Currently ABMs are severely limited by their computational requirements. For instance, the Netlogo models presented here are limited to a few thousand agents running abstract rules on a high-end desktop machine (specifically a Macintosh MacPro Dual-Core Intel 3.0 GHz Xeon with 8 MB of RAM), with the result that a run of 7 days simulated time in the gut-lung axis ABM takes approximately 30 minutes. While scaling up pure ABM models is at this time not feasible, there is promise on the horizon. Advances in supercomputing have moved into implementation of distributed systems, including grid computing, massively parallel machines such as IBM's Blue Gene P, and the use of novel chip technologies such as the Cell^© ^processor (as found in Sony's PS3) and graphical processing units (GPUs). However, despite the computational promise of these hardware platforms, there are still significant hurdles to the efficient implementation of ABM on these distributed systems. Central to these is the latency between intra-processor computational speed and that of node-to-node inter-processor speed. One approach is to improve the efficiency of the computational demands, such as reducing the number of agents that need to be treated individually via "dynamic agent compression" [56] or streamlining the execution of a computationally expensive step, such as a Gillespie algorithm [57]. Another approach is to develop novel load-balancing algorithms, ironically inspired by biological systems, that offer the promise of finding a solution to the challenge of distributing an ABM across a distributed system [58-60]. That a full-scale ABM implementation is not possible at this time does not obviate the need to develop and communicate the potential framework that is conceptually robust and allow the evolution of knowledge represented in a computable form.

In short, the agent-based paradigm, with its defining characteristics of encapsulation, modularity and parallelism, can provide an over-arching design architecture for the computational representation of biological systems. The examples presented herein are intended to be an introduction to this framework. For example, the detail of the molecular events can be represented at a finer grained level using ordinary differential equations, Gillespie-type algorithms or even particle-based signaling models. Cell behavior can be expressed as differential equation models derived from more detailed kinetic knowledge of their response curves. The physiological functions of individual organs can be represented using detailed physical system models detailing shear forces, stress response curves and contractility patterns. Every encapsulated object, at any hierarchy, can be represented in exhaustive detail using mathematical tools. However, two primary question exist: 1) is it even possible to exhaust the level of detail achievable to a pure reductionist's satisfaction? And 2) is it even necessary for the goal of conceptual model verification and representing knowledge? The modular, multi-scale agent-based architecture presented herein does not seek to answer those particular questions, but does hope to function as a seeming paradoxical solution to both questions by: 1) offering the opportunity to dig as deeply and with as much detail as desired, but also 2) to allow knowledge to be expressed effectively and usefully in the qualitative fashion that most researchers use to establish their conceptual models. This latter point cannot be over-emphasized, as ultimately the defining aspect of science is skepticism, the Popperian goal of hypothesis nullification.

## Availability

The software used to create this model, Netlogo [28], is freely available for download at:. Netlogo is a self-contained modeling toolkit, and is available for Windows, Macintosh and Linux. The Netlogo version of the innate immune response/endothelial model can be accessed at . The EBABM itself is available for download at: . The endothelial/inflammatory cell model can be downloaded at: . The Gut ABM and the Gut-Lung = Axis are available for download at: .

## Abbreviations

ABM: Agent based modeling; ARDS: Adult respiratory distress syndrome; Caco-2: culture human enterocyte line; FURM: functional unit representation method; EBABM: epithelial barrier agent based model; ICU: Intensive care unit; IFN-gamma: interferon-gamma; IL-1: interleukin-1; iNOS: inducible nitric oxide synthetase; MOF: Multiple organ failure; NAD^+ ^: nicotinamide adenine dinucleotide; NF-kappa-B: nuclear factor-kappa B, NO: nitric oxide; NSF: N-ethylmaleimide-sensitive factor; SIRS: Systemic inflammatory disress syndrome; TJ: tight junction; TNF: tumor necrosis factor

## Competing interests

The author declares that they have no competing interests.
